# Recommended Practices to Eliminate *Campylobacter* from Live Birds and Chicken Meat in Japan

**DOI:** 10.14252/foodsafetyfscj.D-20-00021

**Published:** 2021-09-24

**Authors:** Asano Kozu Clarke, Said Ajlouni

**Affiliations:** School of Agriculture & Food, Faculty of Veterinary & Agricultural Sciences, The University of Melbourne, Building 142, Royal Parade Melbourne 3010, Australia

**Keywords:** *Campylobacter*, campylobacteriosis, food regulation, food poisoning, HACCP

## Abstract

*Campylobacter* food poisoning is one of the major bacterial foodborne diseases resulting in numerous outbreaks worldwide. Particularly in Japan, one-fourth of the total food poisoning is caused by *Campylobacter jejuni/coli*. Raw and/or undercooked poultry meat and meat products are known as the main cause of campylobacteriosis. Consequently, effective and immediate actions are needed to eliminate or at least reduce campylobacteriosis. This study aimed at examining the Japanese food regulation system, comparing it with those in the USA and Australia, and making necessary recommendations for a better control of campylobacteriosis in Japan. The study was conducted by a thorough investigation of published literatures, governmental documents, statistical and epidemiological data and public information. The results led to recommendations that the Japanese food regulation authority should consider the following suggestions in order to control campylobacteriosis: 1) assess the *Campylobacter* safety at the end of processing stage of chicken supply chain based on risk assessment using quantitative/qualitative baseline data collected over Japan, 2) establish a national *Campylobacter* strategy, including specific campylobacteriosis reduction goals and criteria, and 3) provide the small food business operators with sufficient training and support to implement a Hazard Analysis Critical Control Points (HACCP) as an obligatory food safety requirement. It is acknowledged that it would be difficult to apply foreign regulations directly to Japanese food regulation system due to differences in food culture, regulation, industry structure, and data collection systems. Thus, flexible application is required. Finding and conducting effective *Campylobacter* control measures can decrease contaminated live birds and chicken meat in Japan, home to a unique food culture of eating raw and/or undercooked chicken meat called Torisashi such as sashimi, tataki and yubiki chicken. Consequently, potentially available research data may be instrumental in finding solutions for reducing campylobacteriosis. Eliminating *Campylobacter* food poisoning cases in Japan will be a significant achievement in ensuring Japanese and global food safety.

## 1. Introduction

Foods provide excellent medium and nutrients for the growth of many pathogens and food spoilage microorganisms. Consequently, foodborne diseases are the major concern of public health and socioeconomic costs^1^^)^. For example, in Japan, 28,723 incidents, 524,776 patients and 119 cases of deaths by foodborne diseases have been reported in the past 20 years^2^^,^^3^^)^.

Additionally, in recent years (2014-2018) 1,132 incidents, 19,214 patients and 7 cases of deaths have been reported annually and bacterial foodborne diseases accounted for 40.5% of the total numbers^2^^,^^3^^,^^4^^)^. In 2010, the Ministry of Health, Labor and Welfare (MHLW) indicated that *Campylobacter jejuni/coli (C. jejuni/coli), Salmonella, Clostridium perfringens, Staphylococcus aureus, Clostridium botulinum, Vibrio parahaemolyticus* and Shiga toxin producing *Escherichia coli* (STEC) O157 were the major foodborne bacterial agents in Japan^5^^)^. Among all these pathogenic bacteria, *campylobacters* are gram-negative non spore-forming bacteria with spiral-shaped rods^6^^,^^7^^)^. They are microaerophilic, possessing motility with one or two polar flagella^6^^)^. The reservoir of *Campylobacter* spp. is the intestine of warm-blooded animals, particularly large numbers of poultry^6^^)^. The usual optimum growth temperature is 37 - 42°C and thermophilic *Campylobacter* such as *C. jejuni* and *C. coli* do not grow below 30°C^6^^,^^7^^)^. *Campylobacter* is susceptible to heat, low water activity, UV light and salt^7^^)^. Because of their microaerophilic and thermophilic characteristic, *Campylobacter* cannot multiply outside of warm-blooded livestock or grow in meat products during either processing or storage^8^^)^. However, *Campylobacter* are able to survive in an environment such as in slurries and dirty water for up to 3 months^7^^)^.

*Campylobacter*-contaminated poultry meat is known as a main source of campylobacteriosis, which is the most frequently reported foodborne illness worldwide^9^^,^^10^^)^.

The main cause of *Campylobacter* infection is insufficient cooking or cross-contamination from poultry meat. *C. jejuni* and *C. coli* are known as the most significant and important species in terms of food poisoning and causing human gastrointestinal disease^6^^,^^11^^,^^12^^)^. *Campylobacter* spp. have been the most common source of foodborne disease in Japan, Australia and the USA^6^^)^. The most recent statistics in 2019 revealed that food poisoning cases in Japan by *C. jejuni/coli* represented the largest bacterial foodborne disease, accounting for a quarter of the total cases^2^^)^. The largest case was an outbreak with more than 500 patients, which was caused by undercooked chicken products being served at an outdoor event^2^^)^. That was due to the nature of Japanese original Torisashi culture. Torisashi, raw and/or undercooked chicken meat such as sashimi, tataki, and yubiki chicken, are commonly eaten in southern part of Japan^13^^,^^14^^)^. Sashimi is a sliced raw chicken meat^15^^)^. Tataki is a lightly seared piece of chicken, burned only on its surface^15^^)^. Yubiki is a cooking method by mildly boiling to heat the outer layers only, leaving the inner part rare and pink^12^^,^^13^^)^. In Australia, campylobacteriosis cases contribute to 30% of the foodborne diseases^16^^)^, in contrast the USA has 0.2% cases of foodborne-related death attributed to campylobacteriosis^17^^,^^18^^)^.

This study aimed at thoroughly examining the food regulation guidelines related to the control of campylobacteriosis in Japan. Comparison of such food safety regulations with those of Australia and the USA will help to make necessary recommendations that can improve food safety and reduce campylobacteriosis in Japan.

## 2. Database Sources and Searches

All data and information have been collected from published literatures, governmental documents, statistical and epidemiological data and public information.

English and Japanese literatures were explored via electronic bibliographic databases shown in Table 1.

**Table 1.** Electronic bibliographic databases to search English and Japanese literatures

	Electronic bibliographic databases
English literature	1. Google Scholar ^10,12,15,19,20,24,25,27,28,32,63,41,42,48,60,68,71,81)^ 2. Scopus (Elsevier) ^6,11,13,14,23,40)^ 3. MEDLINE in PubMed ^1,17,18)^ 4. Web of Science ^7^ 5. Commonwealth Agricultural Bureaux International (CAB) Abstracts ^16,37,45)^
Japanese literature	1. J-STAGE ^26,33,35)^ 2. CiNii ^49,59)^

## 3. Campylobacteriosis

Campylobacteriosis includes symptoms such as severe abdominal pain, fever and diarrhea that are characterized as acute, self-limiting watery or bloody consistency^6^^,^^19^^)^. This occurs usually between 2 to 5 days after an ingestion of pathogens and is rarely fatal^1)^. However, the infection with *Campylobacter* can trigger Guillain-Barré syndrome (GBS), which leads to rapid-onset of muscle weakness and paralysis as the peripheral nervous system is damaged. This occurs in approximately 3 in 10,000 campylobacteriosis cases^11^^,^^19^^,^^20^^)^. It has been reported that 1.15 in 100, 000 campylobacteriosis cases were linked to GBS^21^^)^. In fact, the actual numbers of reported GBS patients after *C. jejuni* infection in Japan were 2 in 1990 and 7 in 1991 in Japan^22^^)^. It was suggested that the lipooligosaccharides (LOS) on the surface of the *C. jejuni* may trigger the GBS neurological symptoms^23^^)^. However, only a few molecular epidemiological analysis on *C. jejuni* isolated from humans and chickens have been conducted in Japan^24^^)^. An investigation by Yabe et al^25^^)^ confirmed that *C. jejuni* sequence types (ST) were carried by chickens in Japan. Furthermore, another study by Kitao et al^26^^)^ reported the presence of LOS genes (*cst-II*, *cgtA*, and c*gtB*) associated with GBS in *C. jejuni* strains were isolated from both human and chickens. These findings highlighted the significant relationship between the detection of *C. jejuni* and the possible development of GBS in Japan^26).^*Campylobacter* spp. are known to thrive in the intestine of various animal hosts, particularly birds^27^^,^^28^^)^. Transfer of these bacteria can occur through handling or consumption of food products or water, as well as contact with infected animals^28^^,^^29^^)^. Poultry and poultry meat products are considered the most significant sources of human infections^10^^,^^24^^)^.

Most campylobacteriosis cases occur with raw and undercooked chicken products, although the risk caused by these hazards can be easily removed with proper heat treatment^30^^)^. *C. jejuni/coli* can cause food poisoning when present in very small numbers 200-800 CFU/g^31^^,^^32^^,^^33^^)^. Consequently, reducing the number of these bacteria to < 200 CFU/g is highly imperative for campylobacteriosis control and prevention. Therefore, good hygiene and sanitary practices throughout the food supply chain are required for all relevant stakeholders to decrease and prevent such foodborne illness^9^^)^.

**Fig. 1.**
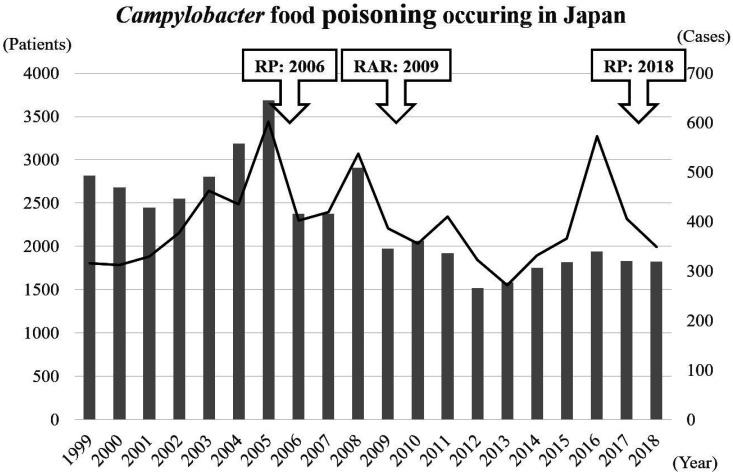
*Campylobacter* food poisoning cases and patient numbers in Japan from 1999 to 2018 Timings of the risk profiles (RP) and the risk assessment report (RAP) publications are also indicated^15^^)^.

In Japan, the consumption of raw chicken meat and the related cases of campylobacteriosis have not decreased enough over the past twenty years (Fig. 1)^15^^)^. Therefore, discussions by the Expert Committee on Microorganisms/Viruses prompted the Food Safety Commission of Japan (FSCJ) to release the following three scientific reports to alert people about the number of campylobacteriosis incidents nationwide^9^^,^^33^^)^.

1. Risk Profile ‘*Campylobacter jejuni*/*coli* in meat products mainly chicken’ for Risk Assessment^34^^)^

2. Risk Assessment Report *Campylobacter jejuni*/*coli* in chicken^33^^)^

3. Risk Profile ‘*Campylobacter jejuni*/*coli* in Chicken Meat and Viscera’ for Risk Assessment^9^^)^

## 4. Information Collection Systems on Campylobacteriosis in Japan

The number of *Campylobacter* food poisoning cases in Japan is usually collected from data via (1) food poisoning statistics based on Food Sanitation Law, (2) Infectious Agents Surveillance Report organized by the Public Health Institute and Public Health Center, (3) infectious gastroenteritis cases collected from medical facilities (13 cities, 16 hospitals) based on National Epidemiological Surveillance of Infectious Diseases^35^^)^.

The collected cases by the health care providers in the public health sectors, according to the restriction of the Food Sanitation Law^36^^)^, only include sick persons who visited hospitals for consultation and provided stool samples that were tested at a clinical laboratory to identify the potential pathogen^37^^)^. Therefore, these passive surveillances results reported to public health officials are limited. According to the active surveillance data studied by Kubota et al^37^^)^, only 32.0% of those possessed acute gastrointestinal illness.

In Japan, the Act on the Prevention of Infectious Diseases and Medical Care for Patients with Infectious Diseases (the Infectious Diseases Control Law) does not include notification category as campylobacteriosis, therefore, the number of campylobacteriosis is not reported^38^^)^.

However, the term ‘campylobacteriosis’ has been used as synonymous with *Campylobacter* food poisoning in Japan. In the Infectious Diseases Control Law, campylobacteriosis is treated as infectious gastroenteritis and required to report in Category V Infectious Diseases^38^^)^.

## 5. Poultry Market and Statistics of *Campylobacter* contamination in Poultry Meat in Japan

The Japanese consumption of poultry meat is mainly comprised of chicken (broiler 90% and spent laying hens 10%), and broiler chickens are known as the main sources of campylobacteriosis^12^^,^^39^^)^. Nationwide, chicken products are normally distributed and sold fresh, while frozen chicken sale is not common^9^^,^^15^^)^.

The statistics on *Campylobacter* rates of infection in chicken meat vary depending on the source of information and chicken processing stages. For examples, (1) the study by Chuma et al^40^^)^ on poultry farms reported the infections rates by *C. jejuni* and *C. coli* in broiler flocks to be 20.0% and 4.7%, respectively, while Ono & Yamamato^41^^)^ indicated that the rate was 75.0%. Yamazaki et al^42^^)^ examined *Campylobacter* from 25 broiler flocks and 9 farms and concluded that the rates were 44.0% and 88.9%, respectively. (2) At the slaughterhouse stage, there were few reports regarding the rates of infection in various chicken meat products during possessing. Data in Table 2 compared the rate of infection in relation to *Campylobacter* positive or negative flocks^43^^,^^44^^)^. (3) At the consumption stage, *Campylobacter* contamination levels of fresh chicken meat in retail and restaurants are limited due to the lack of baseline data and integrated testing protocol^45^^)^. Nevertheless, according to a study by Sasaki et al^43^^)^, *Campylobacter* positive rate in packed chicken products produced from 22 broiler farms was 33% (198/600). Another study by Amano et al^46^^)^ indicated that the skin samples collected from carcasses after evisceration was 100% *Campylobacter* positive and after the chilling processing rate was 80%.

**Table 2.** *Campylobacter* contamination rates on various chicken meat products^43^^,^^44^^)^

Sources of samples	Chicken product	Percentage contamination (Number of cases)	
*Campylobacter* positive flocks	Gizzard and liver	51.1 (180/350)	91 (246/270)
	Thigh	60 (42/70)	-
	Breast	66 (46/70)	99 (89/90)
	Tender	46 (32/70)	74 (67/90)
*Campylobacter* negative flocks	7.2 (18/250)		27% (8/30)

Sources: Sasaki et al^43^^)^ and MAFF^44^^)^

## 6. Causes of *Campylobacter* contamination in Chicken Meat in Japan

According to FSCJ’s (RP) (2018), the collection and understanding of quantitative data in *Campylobacter* contamination levels are not sufficient^9^^)^. The bacterial characteristics make it difficult to control because they are microaerophilic, and the full extent of their environmental resistance such as the viable but not culturable bacteria remains to be investigated^9^^)^. Furthermore, there is little impact on the productivity of chicken farming as chicken flocks can coexist with *Campylobacter*^9^^)^. The non-standardizing quantitative test methodology is also an issue and thus, the baseline data of an entire chicken production chain using the same testing method is not available. The effect of Hazard Analysis Critical Control Points (HACCP) enforcement on chicken contamination has not been assessed either^9^^)^.

### 6.1 Various Stages of Contamination with *Campylobacter*

#### 6.1.1 At the farm

Sources of *Campylobacter* invasion into broiler farms have not clearly been identified^42^^)^, however, main risk factors of high *Campylobacter* prevalence at broiler farms may include water and/or feed supply, environmental bacteria invasion into houses via insects (e.g. flies) and wildlife, washing and disinfection of flocks housing, increasing flock size and a number of flock house, spreading manure on a farm in the winter, farmworkers’ movement among houses, geographical location and seasons^9^^,^^15^^,^^43^^,^^47^^)^. To prevent bacterial transmission at chicken farms, adequate biosecurity measures and hygiene practices are necessary^15^^)^. As these risk reduction measures vary according to each farm environment, such as location, raising system, water and feed supply system and housing structure, farmers find it difficult to design and apply best hygiene practices (biosecurity measures)^15^^)^. Additionally, the rate of spread of *Campylobacter* at farms is significantly rapid among flocks, however, the producers normally do not prioritize the prevention measures against *Campylobacter* exposure as live flocks do not show any infectious symptoms such as decline in productivity^12^^,^^15^^)^. Also, neither economic advantages against *Campylobacter* control nor secured *Campylobacter* eradication measures have been adequately identified by the food businesses operator (FBO) yet.

#### 6.1.2 Slaughterhouse stage

Contamination with *Campylobacter* starts during transportation of live birds to the slaughtering houses. The bird transportation containers are contaminated with fecal deposits, which can contaminate the birds’ feather. The pathogen cannot be removed from the skin completely even after defeathering, and chicken will be sold with this skin^9^^,^^48^^)^. Another source of contamination at slaughtering stage is when the *Campylobacter* positive and negative flocks entered a processing plant together^15^^,^^43^^,^^48^^)^. At the poultry meat processing plants, it is difficult to conduct scheduled slaughtering (logistic slaughtering), since rapid and simple on-site *Campylobacter* detection methods have not been developed^9^^)^. During the slaughtering process, chicken carcasses, particularly their intestines are potentially damaged by defeathering and evisceration leading to leakage or rupture and subsequent *Campylobacter* contamination on carcass^15^^,^^48^^,^^49^^)^. Disinfectant such as sodium hypochlorite (NaOCl) used to eliminate the pathogen from chicken carcass, has low bactericidal effect^15^^)^. Moreover, cross-contamination may occur on the processing line such as cutting or packing due to insufficient washing or sanitation of equipment and handling before each individual carcass processing^15^^,^^48^^)^.

#### 6.1.3 Consumption stage

**Fig. 2.**
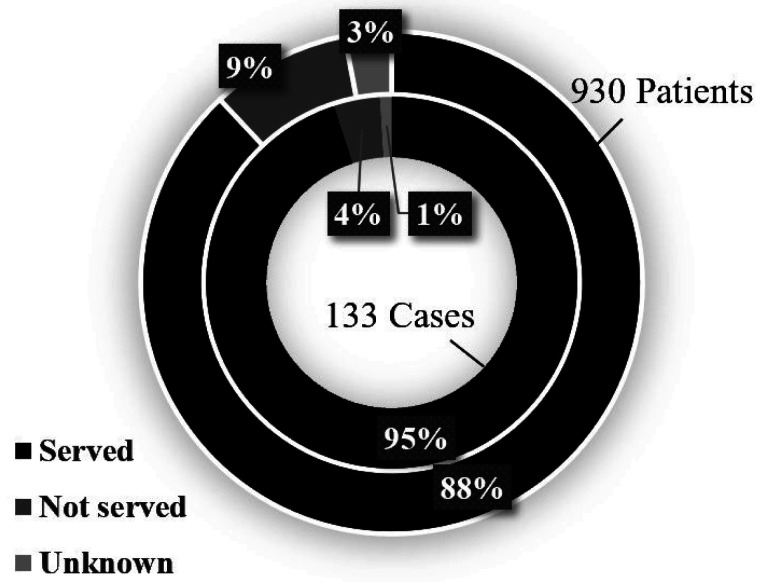
*Campylobacter* food poisoning as a result of raw and/or undercooked chicken servings in Japan^50^^)^ Inner circle: The number and percentage of cases that were confirmed with the fact that raw and/or undercooked chicken meat or offal tissues were served. Outer circle: The number and percentage of patients who were confirmed to have ingested raw and/or undercooked chicken meat or offal tissues.

**Fig. 3.**
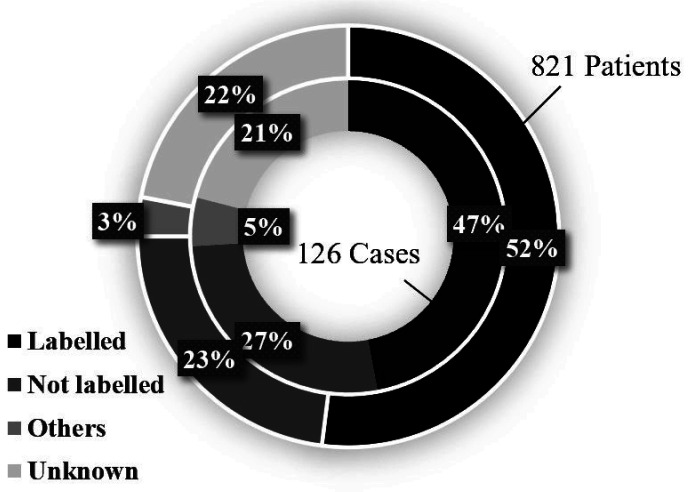
*Campylobacter* food poisoning stemming from raw and/or undercooked chicken meat or offal tissues servings labeled as needing heat treatment in Japan^50^^)^. Inner circle: The number and percentage of cases that were confirmed with the fact that raw and/or undercooked chicken meat or offal tissues were served in spite of labeling as needing heat treatment. Outer circle: The number and percentage of patients who were confirmed to have ingested raw and/or undercooked chicken meat or offal tissues, despite labels of needing heat treatment.

According to the MHLW^50^^)^, 95% of *Campylobacter* food poisoning cases is caused by raw and/or undercooked chicken servings in Japan (Fig. 2). Furthermore, one half of *Campylobacter* food poisoning cases is caused by raw and/or undercooked chicken meat or offal tissues serving even though it was labeled as needing heat treatment (Fig. 3).

Misawa^48^^)^ described that the risk of *Campylobacter* food poisoning could be reduced drastically by perfect implementation of cross-contamination prevention and heating treatment on chicken meat. However, the FBOs and consumer awareness to food poisoning risk from raw and/or undercooked meat as well as cross-contamination at kitchen is very low and *Campylobacter* control on consumption level is difficult unless they stop eating raw and/or undercooked chicken meat^9^^,^^48^^)^.

## 7. Regulation of *Campylobacter Jejuni/coli* in Japan

The food regulation system in Japan is managed by four governmental organizations, namely, FSCJ, MHLW, Ministry of Agriculture, Forestry and Fisheries (MAFF) and Consumer Affairs Agency (CAA)^51^^)^.

FSCJ released a PR for the Assessment of *C. jejuni*/*coli* in Chicken Meat and Viscera in 2018 and notified the MHLW, MAFF and CAA^9^^)^. The Food Sanitation Act authorized by the MHLW was established to prevent food poisoning by selling harmful foods^36^^)^. This act regulates a wide range of FBOs, such as food manufacturing and food import. The act also covers food additives, equipment and containers/packages, which come into direct contact with foods during handling, manufacturing, processing and delivery^36^^)^. Moreover, the Poultry Slaughtering Business Control and Poultry Inspection Law (Japanese Poultry Law) authorized by the MHLW has been involved in public health to avoid poultry meat contamination and to ensure meat safety at slaughterhouses and processing plants^52^^)^. This law established the governmental responsibilities and outlined the licensing and obligation of FBOs, e.g. slaughterhouse, and poultry inspection targeting chickens, ducks and turkeys^52^^)^. The Japanese poultry inspection is structured in accordance with the scale of poultry slaughterhouses (Table 3)^15^^,^^52^^)^.

**Table 3.** The poultry slaughtering system in Japan^15^^,^^52^^)^

Categories	Large-scale facilities	Small-scale facilities
Scale of slaughterhouses	Slaughterhouses processing over 300,000 poultry annually	Slaughterhouses processing less than 300,000 poultry annually
Numbers of facilities in 2017	146	1,776
Duties of poultry meat inspection	Veterinarian should inspect individual birds	Licensed sanitation supervisors can inspect
The difference in processing styles	‘Nakanuki’ method: (Automatic evisceration using machine is conducted before chilling and cutting)	‘Sotohagi’ method: (All muscle parts are removed from the carcass before evisceration)

MHLW monitors domestic and imported food safety guidelines, plans, and inspection measures and imposes penalties for non-compliance^51^^)^. The structure and role of the MHLW are executed by the headquarters, the regional bureaus of health and welfare, and the quarantine stations^51^^)^ as shown in Table 4. Corporates also work with the local governments to ensure proper food safety measures related to food poisoning cases^51^^,^^53^^)^.

**Table 4.** Role of various Japanese organizations in risk analysis and controlling food safety^51^^)^

Related Governmental organization	FSCJ	MHLW	MAFF	CAA
Principal responsibility	Science-based risk assessment of food safety risks to human health	Risk management related to food hygiene	Risk management related to agriculture and forestry, livestock management, and fisheries	Risk management related to labeling of food items
Common responsibility		Risk communication		
Food regulation acts	Food Safety Basic Act	Food Sanitation Act, Poultry Slaughtering Business Control and Poultry Inspection Law etc	Agricultural Chemicals Control Act, Feed Safety Act, etc	Food Labeling Act, Health Promotion Act, etc

The local governments establish their own Food Safety Ordinance, implement the Food Safety Promotion Plans and coordinate among relevant departments to ensure food safety from farm to plate^54^^)^. Additionally, each local government will plan annual monitoring control based on the Food Sanitation Act to prevent food-related poisoning. Furthermore, they usually hold workshops for FBOs and consumers to demonstrate proper meat handling. They distribute leaflets to raise consumer awareness of food poisoning risk caused by raw and/or undercooked meat including chicken, beef liver and pork^55^^)^.

Although multiple Japanese governmental institutions including FSCJ, MHLW, CAA, MAFF and the local governments have continually reported the health risks associated with raw and/or undercooked chicken^56^^,^^57^^)^, the current Japanese Food Sanitation Act does not have standards for restricting raw chicken consumption^15^^,^^58^^)^.

### 7.1 Local Torisashi Regulation in Miyazaki and Kagoshima Prefectures

Prefectural governments operate poultry meat inspection, licensing by confirming compliance and provide food safety related advice to FBOs based on Japanese Poultry Law^52^^,^^59^^)^.

In Kagoshima and Miyazaki prefectures, located in southern Japan, also known as the Kyusyu area, many FBOs commonly sell edible raw and/or undercooked chicken meat, called “Torisashi”. The local governments in these prefectures are trying to establish their own special guidelines for these chicken products to ensure their unique Torisashi safety^15^^)^. This regulated edible raw and/or undercooked chicken meat will be referred to as ‘regulated Torisashi’ in this study.

These guidelines include standards of composition, processing, cooking, storing conditions, equipment, containers and labeling of packages. These standards aim at ensuring the safety of edible raw and/or undercooked chicken meat, in accordance with the enforcement of the Japanese Poultry Law^52^^,^^60^^,^^61^^)^. For example, according to the compositional standards, fecal coliforms, the genera *Salmonella* and *Campylobacter* and the bacterium *Staphylococcus aureus* must not be detected^60^^,^^61^^)^. Consequently, each farm must submit to the slaughterhouse the test results of bacterial agents causing foodborne disease in chicken before bringing broiler flocks, and slaughterhouses to confirm the meat adequacy for the use of regulated Torisashi^61^^)^.

Additionally, chicken vendors and buyers should confirm whether or not their transaction is for regulated Torisashi^61^^)^. For the processing of regulated Torisashi, designated space restricted from other sectors should be prepared^60^^)^, and processing tools such as cutting boards and knives should be exclusively use for of chicken meat^60^^,^^61^^)^. Chilled regulated Torisashi products should be stored at <10℃^60^^,^^61^^)^ and frozen products at <−15℃, or preferably −18℃^55^^)^. When serving to consumers, FBOs must not contain regulated Torisashi on the menu, and it can only be provided at consumer’s request^61^^)^. Additionally, they need to inform customers that regulated Torisashi may carry the risk of food poisoning and vulnerable people such as children and the elderly need to avoid its consumption^60^^,^^61^^)^. The compliance to the compositional standards should be tested voluntarily via registered and qualified laboratories more than twice per year^61^^)^. Furthermore, the labeling of regulated Torisashi should clearly indicate the processing information such as purpose of the chicken consumption ‘Processed for regulated Torisashi’, the location and name of slaughterhouse and processing plants, and the possible risk of regulated Torisashi^60^^,^^61^^)^.

A study by Kakiuchi et al^58^^)^ indicated that *Campylobacter* food poisoning caused by Torisashi, processed and sold in Kagoshima was much lower than those in most metropolitan areas, and in 2017 they had no *Campylobacter* food poisoning cases^58^^)^.

### 7.2 HACCP Principles for All Food Business Operators in Japan

**Fig. 4.**
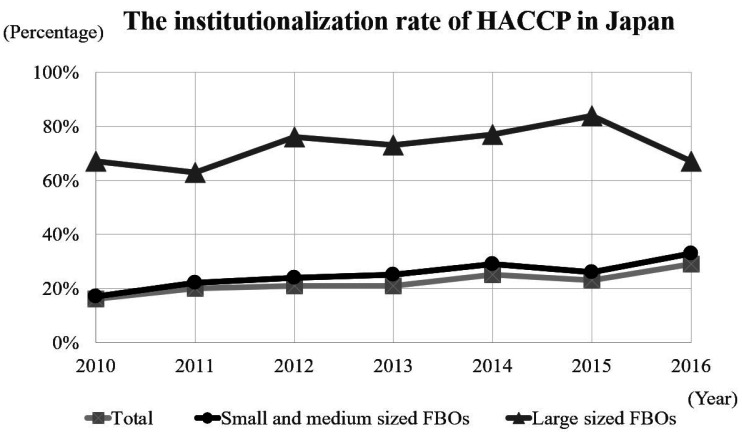
The rate of HACCP adaptation in Japan between 2010 and 2016^53^^,^^63^^)^ Small- and medium-sized FBOs: food business operators with more than 100 million yen and less than 5 billion yen. Large-sized FBOs: food business operators whose food sales are over 5 billion yen.

Japan has established a comprehensive sanitary control system in 1995, based on HACCP^62^^)^. This HACCP system allowed the MHLW to give approval to individual FBOs via audits of manufacturing or processing methods of the target foods, including meat products, and sanitary-control methods. However, small to medium sized FBOs did not adequately introduced or adapted HACCP system as illustrated in Fig. 4^53^^,^^63^^)^.

Therefore, MHLW decided to promote the food hygiene control, based on the Codex Alimentarius HACCP seven principles. Consequently, all abattoirs and poultry processing plants were required to follow the Codex guideline. As for poultry processing plants, MHLW introduced a more flexible HACCP system in 2014. However, the study conducted by Vetchapitak^15^^)^ in 2019 showed that only 23.1% of large-scale poultry processing facilities had introduced the HACCP system.

## 8. Campylobacteriosis in Australia (Information Collection Systems)

Campylobacteriosis will be reported to the state or territory health authorities and managed under each jurisdictional legislation^64^^)^. The data regarding campylobacteriosis will be sent to the National Notifiable Disease Surveillance System (NNDSS) under their public health legislation^58^^,^^59^^,^^60^^,^^61^^,^^62^^,^^63^^,^^64^^,^^65^^)^. The NNDSS collates the gathered data nationally and reports to OzFoodNet^64^^)^. The OzFoodNet is an Australian epidemiological network organized by the Commonwealth Department of Health to manage human foodborne diseases surveillance by identifying their causes and finding risk reduction measures^64^^,^^66^^)^. When an outbreak occurs, the information will be delivered to State Food Safety regulators including other relevant state governmental departments and local government authorities for a coordinated investigation^64^^,^^65^^)^.

### 8.1 Statistics of *Campylobacter* contamination in Poultry Meat and Campylobacteriosis in Australia

According to the study of Walker et al^67^^)^, the recent *Campylobacter* contamination level of retail chicken meat in Australia was normally low, despite their high prevalence on chicken meat, such as 84% in New South Wales (NSW), 90% in Queensland (QLD), and 96% in Victoria (VIC)^67^^)^. In that study, 552 chicken meat samples in total including the following meat portion (Breast:117, Drumstick:102, Marylanda:54, Thigh:106, Wing:84, Whole:89) were collected from retail outlets in NSW, QLD, and VIC from 2016 to 2018^67).^ The quantitative analysis revealed that 98% of chicken meat samples were contaminated with <10,000 CFU *Campylobacter* per carcass, which was below the *Campylobacter* criteria shown in the Australian national guideline, with the most common species detected in chicken meat being *C. coli*^67^^)^.

**Fig. 5.**
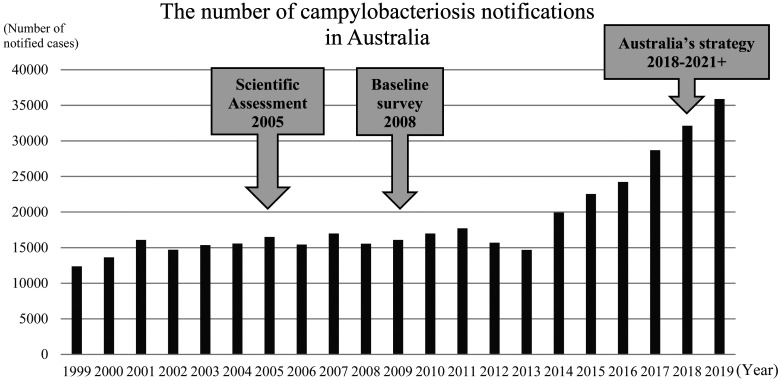
The number of campylobacteriosis notification in Australia^68^^)^

According to the NNDSS of Australian government, Department of Health^68^^)^, the number of campylobacteriosis notifications in Australia has been reported as shown in Fig. 5^68^^)^. However, these total numbers of campylobacteriosis do not describe the human illness cases caused by food source, particularly chicken meat in Australia. Hall et al mentioned that the food source attribution of campylobacteriosis in Australia was 75%^69^^)^. A risk assessment by FSANZ (2005) identified poultry meat as the main source of campylobacteriosis cases in Australia^70^^)^, however, quantitative data were not enough to estimate the chicken meat attribution within total campylobacteriosis cases.

### 8.2 Food Regulation Related to *Campylobacter Jejuni/coli* in Australia

In Australia, the approach and control against hazardous agents, source of foodborne diseases are regulated by the Food Standards Code (The Code) established by Food Standards Australia and New Zealand (FSANZ)^64^^,^^71^^)^. FSANZ is an independent statutory authority, set under the Food Standards Treaty between Australia and New Zealand^71^^)^. However, excluding the Federal Parliament, Australian legislation for food regulation is structured from the eight parliaments of the States and Territories^71^^)^. Each state and territory therefore manage food safety based on their own food acts within their respective jurisdictions^71^^)^. To ensure a nationally consistent approach for the food standards’ implementation and enforcement, the Food Regulation Standing Committee (FRSC) coordinate the policies and pass them to the Australia and New Zealand Ministerial Forum on Food Regulation^72^^)^. The members of the FRSC are senior officials of relevant government departments such as Health, Primary Industries, and Consumer Affairs^72^^)^.

#### 8.2.1 Poultry regulation requirements in Australia

The regulations for poultry meat safety are covered by the Codes in the Food Safety Standards (Chapter 3 – Australia only); the Primary Production and Processing Standards (Chapter 4 – Australia only) and the Microbiological Limits for Food (Standard 1.6.1). These Codes had included a national control measures against bacterial pathogen on a whole chicken supply chain. The codes required the production and processing of safe poultry meat as the Primary Production and Processing Standard for Poultry Meat (Standard 4.2.2)^64^^)^. This poultry standard is applied only in Australia and aims at lowering the *Campylobacter* prevalence and levels in poultry meat^73^^,^^74^^)^.

Further legislation requirements for poultry meat safety include ‘the Australian Standard for Construction of Premises and Hygienic Production of Poultry Meat for Human Consumption based on HACCP principles’^64^^,^^75^^)^.

#### 8.2.2 Criteria for assessing Campylobacter safety in Australia

The main criteria that are used for measuring food safety in Australia include (1) Microbiological criteria for ready-to-eat foods, as described in the Code, and (2) Process hygiene criteria, which are listed in the Compendium of Microbiological Criteria (CMC) for Food.

According to the CMC, *Campylobacter* spp. should not be detected in 25 g food samples^76^^)^. If detected, the decision to dispose or recall the food products from the same lot of the test sample is made along with the mandatory sample investigation and assessment of the incident^76^^)^. Frozen food samples should not be tested because *Campylobacter* count decreases at freezing temperatures and results will not be accurate^76^^)^. Furthermore, the validation of poultry meat safety requires that the total count of *Campylobacter* to be <10,000 CFU per whole chicken carcass at the final stage of processing^76^^)^.

#### 8.3 Australia foodborne illness reduction strategy 2018-2021+

Campylobacteriosis via foodborne transmission has remained high in Australia (e.g., 234,000 cases, including 3,200 hospitalizations and 3 deaths in 2010) compared to the USA, Canada, the UK and the EU^77^^)^. Therefore, the Australian government decided to prioritize reducing this foodborne disease and developed an Australian strategy, ‘Food Regulation Priorities 2017-2021: Reducing foodborne illness, particularly related to *Campylobacter* and *Salmonella*^73^^,^^77^^)^. This strategy was established to decrease the number of campylobacteriosis related to food in Australia by using more quantitative measures by 2021, and to focus on epidemiological and surveillance information and data regarding campylobacteriosis^77^^)^. To achieve the goal of this strategy, it is necessary that all stakeholders, from farm to consumers, corporates and participate in the whole food supply chain^77^^)^. Thus, FRSC, the entity to implements the strategy and informs the Forum of updates, assisted discussion among industry, public health and consumer stakeholders for the development of strategy^77^^)^.

In 2007, the Australia and New Zealand Ministerial Forum on Food Regulation supported the Food regulation system to produce a strong food safety system that can improve food safety and reduce salmonellosis and campylobacteriosis from 2018 to 2021 and beyond (2021+). The strategy is comprised of the following six elements in 2007, as in **Table 5**^77^^)^. (1) National engagement, (2) sector based initiatives, (3) research, (4) monitoring and surveillance, (5) consumer and industry information, and (6) food safety culture.

**Table 5.** The six key participants in the Australian Food-borne Illness Reduction Strategy 2018- 2021+^77^^)^

Key participants of the Australian strategy	Responsibility
1. National engagement	Sharing activities and outcomes nationally, establishing industry and government forums (specifically in poultry sector)
2. Sector based initiatives	Implementation of the strategy, reviewing of poultry meat production and processing standard
3. Research	Taking actions based on research and evidence, and sharing the knowledge among governments, industry and researchers
4. Monitoring and surveillance	Let governments, industry and consumers choose and implement best public health and safety action, and provide comprehensive, integrated and systematic data, nationally surveyed and monitored
5. Consumer and industry information	Improve the guidance and education to industry and consumers, resourcing necessary and consolidated information about food business knowledge, practices, awareness and commitment to food safety culture, for education and training providers
6. Food safety culture	Promote and improve food safety culture among various stakeholders nationally, focusing on food safety culture by upskilling of governmental officials, working with educational institutions, monitoring industry’s food handling behaviours, and enhancing consumer knowledge through resources and training

#### 8.3.1 Australian Campylobacter reduction strategy

The focuses of *Campylobacter* risk assessment were set as food safety risk associated with the consumption of poultry meat and poultry meat products in Australia. The strategy also examines the factors that have the greatest impact on public health and safety throughout the poultry meat supply chain^70^^)^. This RAR aimed at giving scientific evidence to develop Standard 4.2.2 in Australia and to prescribe appropriate risk management measures to protect consumers from foodborne illnesses from poultry meat consumption^70^^)^.

That risk assessment was based on the FAO/WHO’s risk assessment model. The quantitative assessment for *Campylobacter* in chicken meat was implemented only from processing plants to consumption level on food supply chain^70^^)^. The assessment at farm level was not included and estimating quantitative risk association with various practices conducted on farm was not possible, either^70^^)^. According to the risk assessment, 93% of the estimated number of campylobacteriosis cases will be decreased if a ten-fold reduction could be achieved at the end of processing stage^70^^)^.

The risk assessment concluded that various factors have been reported as influence to the contamination possibility in processing plants and during distribution, handling, preparation and consumption stages^70^^)^. (Table 6)

**Table 6.** Effect of various processing stages on *Campylobacter* contamination level on chicken carcasses^70^^)^.Order of process stage and its effects on contamination by *Campylobacter*

1. Stun/Slaughter		Minimal	
2. Scald-Low temperature	Reduce		
3. Scald-High temperature	Reduce		
4. Defeathering			Increase
5. Washing	Reduce		
6. Evisceration			Increase
7. Washing	Reduce		
8. Chilling (immersion)		Minimal	
9. Chilling (air)		Minimal	
10. Portioning		Minimal	

In general, the number of contaminated chickens increases while chickens are transported from farms to slaughterhouse. However, the contamination level on chicken carcasses decreases when processed^70^^)^. The bacterial prevalence normally increases after evisceration and decrease at chilling stage with effective operation^70^^)^. Influences of the possible campylobacteriosis occurrences are the contaminated pathogen level in poultry meat and prevalence at the end of processing and cross-contamination opportunities at handling and preparation stage^70^^)^.

## 9. *Campylobacter* in the USA

The national baseline data on chicken meat was collected by the Food Safety and Inspection Service (FSIS) within the Raw Chicken Parts Baseline Survey (RCPBS) in 2012. The survey involved 2,496 samples of various chicken parts produced at 449 establishments^78^^)^. The survey was statistically built to evaluate the entire chicken industry by assessing each establishment, according to their production scale^78^^)^. Results showed that *Campylobacter* positive percentage of skin-on chicken parts samples were significantly higher (24.0%) than skin-off samples (16.6%) and chicken parts were contaminated twice in comparison with a whole chicken^78^^)^. These findings indicated the possibility that pathogens on a single positive carcass were cross-contaminating other chicken parts through processing and handling operation^78^^)^. This national baseline survey result regarding *Campylobacter* was utilized for developing microbiological criteria for industry performance standards of raw chicken parts, considering the difference of regional prevalence^78^^)^.

### 9.1 Statistics of *Campylobacter* contamination in Poultry Meat and Campylobacteriosis in the USA

**Fig. 6.**
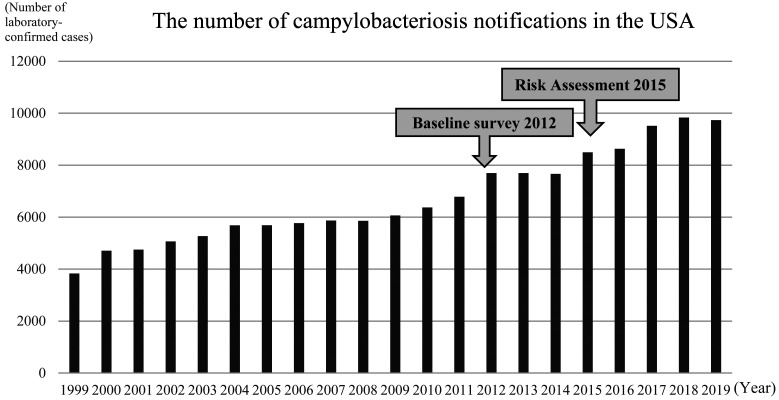
The number of campylobacteriosis notifications in the USA^80^^)^

In the USA, the Interagency Food Safety Analytics Collaboration, which was created from three institutions, (1) the Center for Disease Control and Prevention (CDC), (2) the Food and Drug Administration (FDA) and (3) the FSIS, estimated the sources attribution of campylobacteriosis based on 236 *Campylobacter* outbreaks data data 1998 - 2017)^79^^)^. The 147 outbreaks attributed were excluded from the estimation because they were dairy-based cases. The remaining 89 *Campylobacter* outbreaks were used for the analysis of chicken-related campylobacteriosis outbreaks^79^^)^. The number of campylobacteriosis notifications in the USA with no association with outbreaks ranged from about 4,000 to 10,000 in 1999-2019. These yearly case reports of campylobacteriosis were collected from the data of the Foodborne Diseases Active Surveillance Network (FoodNet)^^80^)^ in Fig. 6.

### 9.2 Food Regulation Related *Campylobacter Jejuni/coli* in the USA

Following the Federal Meat Inspection Act of 1906, the Poultry Products Inspection Act has regulated the control of contamination in poultry meat since 1957 in the USA^78^^,^^79^^,^^80^^)^. Meat inspection was identified as a compulsory requirement to ensure consumers’ safety.

Subsequently, the HACCP system was introduced in the USA by FSIS in the United State Department of Agriculture (USDA)^39^^)^. The role of FSIS is to ensure the safety of meat, poultry, and egg products, including their labeling and packaging. FSIS conducts regular inspection and monitoring, and verifies the appropriate processing, handling, and labeling from the primary chicken production to the consumption^81^^)^. They cooperate with FDA and states agencies^39^^)^. The state authorities normally follow the federal legislation. However, they can also establish their own food-related laws and policies^82^^)^. As well as at the state level, the local government can develop their original laws or policies according to the regional characteristics or needs, following the federal or state regulation guidelines^82^^)^. Additionally, CDC contributes to assessment of research data regarding industry progress to reduce product contamination and foodborne disease caused by poultry meat^83^^)^.

Concerning poultry regulation, USDA is traditionally in charge of producing the livestock to be slaughtered at abattoirs and processed products in sanitary condition, both in state and interstate levels^39^^)^. Therefore, they are responsible for identifying and eliminating potential food safety risk and hazards existing in the production facilities^39^^)^. These establishments are required to be inspected by FSIS, which issues numerous product control and enforcement measures for consumers’ safety and confirm any violations of the law^39^^)^. Once the products pass inspection by FSIS, the official mark of USDA will be given so that FBOs can sell them in interstate commerce^39^^)^.

### 9.3 HACCP Legislation of Poultry Meat and Information Collection Systems of Campylobacteriosis in the USA

The mandatory HACCP requirements (HACCP Systems Final Rule: 61 FR 38806) of meat and poultry manufacturing was introduced in 1966 to the USA^84^^)^. However, effective and successful HACCP plan requires implementation of both Standard Operating Procedures (SSOPs) and Good Manufacturing Practices (GMPs)^85^^)^. The SSOPs are defined in the Federal Meat Inspection (9 CFR 416) or State Meat Inspection programs^85^^)^, and the GMPs are specified in the regulations for meat and poultry operations.

Various agencies contribute to the campylobacteriosis information collection. Since 2015, NNDSS has been actively recording the number of campylobacteriosis^86^^)^. In 1996, the Food-borne Diseases Active Surveillance Network (FoodNet) started and continue the campylobacteriosis active surveillance^86^^)^. The National Outbreak Reporting System helps reporting the outbreak information to CDC surveillance systems^86^^)^.

## 10. Common Applied Strategies in Australia and The USA to Control *Campylobacter*

As discussed in sections 8 and 9, both Australia and the USA have implemented a national baseline surveys to collect data before or after conducting *Campylobacter* arisk assessment^70^^,^^74^^,^^78^^,^^87^^)^. The common trends to set *Campylobacter* regulations in these two countries involve: 1) mentioning the importance of *Campylobacter* control on the entire chicken supply chain; 2) Focusing on the sample collection at the end of processing stage to evaluate the *Campylobacter* prevalence on chicken meat; 3) utilizing the baseline data from risk assessments to develop certain standards regarding poultry meat; and 4) establishing campylobacteriosis reduction goals in risk assessments as summarized in Table 7^74^^,^^78^^,^^87^^,^^88^^)^. Similarly, in 2009, the Japanese food authority has conducted risk assessment for *C. jejuni*/*coli* in chicken^33^^)^. However, there was no available national baseline data tested by the standardized protocol for comparison, particularly quantitative data on the Japanese whole chicken supply chain.

**Table 7.** Comparison between the Australian and the USA baseline survey and risk assessment^68,72,81,82)^

National baseline survey	Australia	The USA
Implementation year	2008	2012
Collected samples	(1) Farm: 233 pooled faecal samples (2) Prior to processing: 636 faecal samples (3) Post processing: 1112 carcass rinse samples	(1) End of the production line: 2,496 chicken parts samples from 449 sites
Utilization of the baseline survey data	To develop the PPP Standard 4.2.2	To establish Campylobacter criteria for industry performance standards in the USA
Risk assessment		
Implementation year	2005	2015
Purpose of risk assessment	To develop the PPP Standard 4.2.2	To establish Campylobacter criteria for industry performance standards in the USA
Campylobacteriosis reduction estimation or goal	Estimation: Ten-fold prevalence reduction at the end of processing stage will lead 93% reduction of the number of campylobacteriosis	Goal ‘The Healthy People 2020’: Achieving 33% reduction of the number of campylobacteriosis by meeting the 50% of compliance fraction in raw chicken parts (Target of the number of food-borne campylobacteriosis: 8.5 per 100,000 population)

**Fig. 7.**
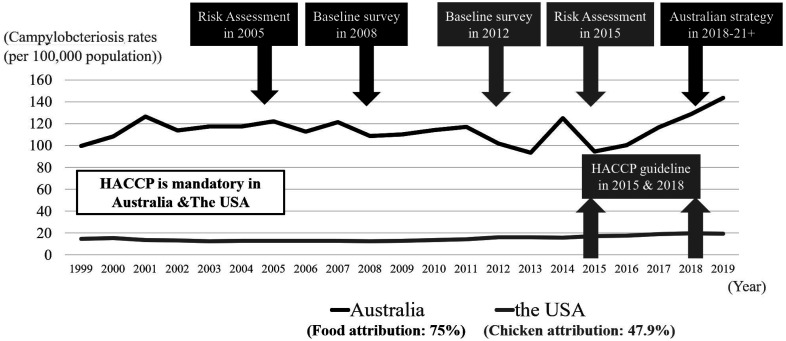
The number of campylobacteriosis rates (per 100,000 population) and *Campylobacter* reduction strategy in Australia and the USA^68^^,^^80^^,^^89^^)^.

Due to the high number of campylobacteriosis in Australia as compared to other developed countries, such as the USA (Fig. 7)^68^^,^^80^^,^^89^^)^, the government started to build a national strategy in 2005 to prioritize decreasing this campylobacteriosis^77^^,^^89^^)^. The Australian strategy aimed at stepping up efforts in collecting epidemiological and surveillance information and data by using more quantitative measures and cooperation among all stakeholders from farm to table, such as authorities, poultry industry, researchers, educational institutes and consumers^77^^)^.

Recently, the USA also established ‘The Healthy People 2020’ as a national goal to reduce the number of campylobacteriosis in the USA^87^^)^. FSCJ released the latest scientific report, RP “*Campylobacter jejuni*/*coli* in chicken meat and viscera” for Risk Assessment in 2018 and made it available to relevant risk management organizations seeking their cooperation to decrease *Campylobacter* food poisoning cases. Nevertheless, there is currently no national strategy and specific numeric goal focusing on campylobacteriosis reduction in Japan similar to those already developed in Australia and the USA.

Apparently, the Japanese food authority has not enforced any national criteria for assessing *Campylobacter* safety similar to those in Australia (the process hygiene criteria of *Campylobacter)* or in the USA (*Campylobacter* performance standards). Consequently, a study by Connerton and Connerton (2017)^6^^)^ showed that, as of year 2017, the frequency of campylobacteriosis was 1,512 cases per 100,000 population in Japan (Fig. 1), which was much higher than those in Australia (112 per 100,000) and the USA (20 per 100,000) (Fig. 7)^68,80,89).^ Both Australia and the USA have already implemented good poultry supply chain regulations following certain *Campylobacter* safety standards.

Therefore, considering these observations, it is recommended that the Japanese food regulation should follow similar approach to what has been enforced in Australia and the USA and apply the following *Campylobacter* reduction strategies:

• Implementing baseline survey at the national level with standardized *Campylobacter* testing methods, desirably, on an entire chicken supply chain.

• Using collected quantitative and qualitative data for risk assessment to set *Campylobacter* standards, particularly at the end of a processing stage.

• Setting specific campylobacteriosis reduction goals to achieve.

Lastly, in order to implement these strategies effectively and comprehensively, the Japanese government should construct a national strategy against *Campylobacter* and show their high awareness of prioritizing this *Campylobacter* issue to all stakeholders in Japan. To run the national strategy, Japan should also create a platform where all stakeholders of the chicken supply chain can cooperate by sharing knowledge and the latest information, and by discussing current issues actively, so that each member can take an initiative in their own sectors (governments, industry, researchers, educational institutes and consumers).

### 10.1 Promoting HACCP Introduction into Small Poultry Businesses

The HACCP system has been an obligatory requirement in Australia and the USA, however, Japan has only recently changed it from a voluntarily to mandatory requirement for all FBOs (section 2.3.3, 2.4.2, & 2.5.2)^53^^,^^75^^,^^84^^)^. In 2015, the HACCP introduction rates in small to medium-sized FBOs of the poultry sectors was 26%, and only 23.1% in large-scale poultry processing facilities (section 2.3.3)^15^^,^^53^^)^. Therefore, the amendment of the current Food Sanitation Act and HACCP enforcement in Japan are essential to significantly improve the poultry meat safety practices at all relevant FBOs. However, many FBOs, particularly small ones, are still not familiar with this system, and require governmental support. Vetchapitak and Misawa^15^^)^ mentioned that the introduction of HACCP can improve the FBOs’ awareness for better food safety practices. Thus, to promote HACCP introduction into small poultry business, the USA’s strategy, which prepares sufficient support such as guidelines for very small and small FBOs, will be useful. As indicated in section 2.3.3, the Japanese regulation has already started to build similar supports for such small FBO. Fig. 3shows, the importance to continually provide such assistance for small FBOs^^50^)^ .

## 11. Conclusion and Recommendations for Controlling Campylobacteriosis in Japan

The aim of this study was to make necessary recommendations to improve the Japanese food regulations and decrease *Campylobacter* food poisoning occurring in Japan. From the findings obtained in this review, it is recommended for the Japanese government to: (1) develop a national strategy against *Campylobacter*, including specific campylobacteriosis reduction goals and criteria assessing *Campylobacter* safety at the end of processing stage, based on the risk assessment using quantitative/qualitative baseline data collected over Japan, and (2) continually promote HACCP introduction into small FBOs as a mandatory food safety requirement by preparing sufficient support for them.

However, there are also some limitations of introducing Australian and the USA *Campylobacter* reduction strategies into Japanese regulation. Such limiting factors are caused by the differences in the food culture (e.g., Torisashi in Japan), the poultry regulation systems, the industry structure and in the data collection systems among the three countries. It is therefore difficult to directly apply the regulation of the two countries (Australia and the USA) into Japanese policy. Flexibility and gradual application are required.

Finding and conducting effective *Campylobacter* control measures can decrease highly contaminated live birds and chicken meat in Japan. These can ease the socioeconomic damage from *Campylobacter* food poisoning incidents and provide consumers with safer chicken not only in Japan, but worldwide. Japan has its unique food culture of eating raw and/or undercooked chicken meat, thus, potentially available research data may be more informative compared to other countries. Eliminating a quarter of all food poisoning cases in Japan will be a significant achievement in ensuring Japanese and global food safety.
